# Retraction: Fortunellin-induced modulation of PTEN by miR-374a decreases inflammation and maintains intestinal barrier function in colitis

**DOI:** 10.3389/fimmu.2026.2002557

**Published:** 2026-09-25

**Authors:** Frontiers Editorial Office

The journal retracts the 26 January 2018 article cited above.

Following publication, concerns were raised regarding the integrity of the images in the published figures. During the investigation, which was conducted in accordance with Frontiers’ policies, the authors provided raw data and additional supporting materials. However, the materials provided were not sufficient to satisfactorily resolve the concerns raised regarding the published figures. As a result, the editors no longer have confidence in the findings presented in the article, and the article has been retracted.

This retraction was approved by the Chief Editors of Frontiers in Immunology and the Chief Executive Editor of Frontiers. The authors were informed of this retraction and agree with it.

Frontiers would like to thank the users on PubPeer for bringing the published article to our attention.

